# *In vivo* Administration of Atorvastatin Does Not Impair Mitochondrial Function or Cellular Viability in the Prefrontal Cortex of Mice

**DOI:** 10.1007/s12640-026-00805-2

**Published:** 2026-06-26

**Authors:** Karen B. Gessler, Júlia Rescaroli, Silvana M. Pires, Francisco G. W. Lippert, Eduarda S. Spanamberg, Gabriel Bernardes, Isabel M. Zavorne, Carla I. Tasca, Gianni Mancini

**Affiliations:** 1 Laboratório de Neuroquímica-4, Departamento de Bioquímica, Universidade Federal de Santa Catarina, Trindade, Florianópolis, SC 88040-900 Brazil; 2 Programa de Pós-graduação em Bioquímica, Universidade Federal de Santa Catarina, Trindade, Florianópolis, SC 88040-900 Brazil; 3 Programa de Pós-graduação em Neurociências, Centro de Ciências Biológicas, Universidade Federal de Santa Catarina, Trindade, Florianópolis, SC 88040-900 Brazil; 4 Universidade do Sul de Santa Catarina, Cidade Universitária, Pedra Branca, Palhoça, SC Brazil

**Keywords:** Atorvastatin, Mitochondria, Prefrontal cortex, High-resolution respirometry, Oxidative stress, Bioenergetics

## Abstract

Atorvastatin is a widely prescribed statin used for its cholesterol-lowering effects; however, it also exerts known pleiotropic effects, including antioxidant and anti-inflammatory properties. Atorvastatin´s impact on mitochondrial function remains controversial, with reports of toxicity in skeletal muscle and liver, while exhibiting beneficial effects on the brain. Atorvastatin has a neuroprotective effect, modulating glutamatergic transmission and reducing oxidative stress. Additionally, we previously showed that atorvastatin treatment in vivo increases mitochondrial capacity in the mouse hippocampus. Nonetheless, the bioenergetic impact of atorvastatin on the prefrontal cortex, a critical region for cognitive function, remains underexplored. This study investigated whether short-term administration of atorvastatin alters mitochondrial respiration and redox status in the prefrontal cortex of adult mice. Male Swiss mice received atorvastatin (10 mg/kg/day) or vehicle orally (p.o., in a voluntary consumption protocol) for 7 days. A behavioral analysis showed that atorvastatin slightly increased spontaneous locomotor activity and does not compromise short- (90 min) and long-term (24 h) memory in the object recognition task. The evaluation of cellular viability, cell membrane integrity, reactive oxygen species (ROS) production, and mitochondrial membrane potential in slices from the prefrontal cortex showed that atorvastatin did not induce oxidative stress, cell damage, or mitochondrial depolarization. High-resolution respirometry (HRR) was used to assess oxygen consumption rates (OCR) in prefrontal cortex homogenates, and we observed no significant alterations in any of the mitochondrial respiratory states: basal, LEAK, phosphorylating, maximal electron transfer system (ETS) capacity, or coupling efficiency. Furthermore, direct analysis of the enzymatic activity of respiratory chain complexes I and II also showed no alterations. These findings indicate that short-term atorvastatin administration is bioenergetically neutral in the murine prefrontal cortex under basal conditions, and does not compromise cognition, mitochondrial function, or cellular viability.

## Introduction

Atorvastatin is a widely prescribed statin that inhibits 3-hydroxy-3-methylglutaryl coenzyme A (HMG-CoA) reductase activity, a key enzyme in the mevalonate pathway of cholesterol synthesis. It is primarily used for its cholesterol-lowering effects and for cardiovascular risk reduction (Zhao et al. 2015; Grundy et al. 2019). In addition to its lipid-lowering effects, atorvastatin exhibits pleiotropic actions such as antioxidant and anti-inflammatory effects (Profumo et al. 2014; Marques et al. 2018b). Beyond that, effects on the central nervous system (CNS) have also been reported, with atorvastatin presenting antidepressant effects in preclinical and clinical studies (Ludka et al. 2013, 2017b; Haghighi et al. 2014), and neuroprotective actions in preclinical models of neurodegenerative diseases, as Alzheimer´s, Parkinson´s, traumatic brain injury, and brain ischemia (Bösel et al. 2005; Gutierrez-Vargas et al. 2014; Massari et al. 2016; Mancini et al. 2020). However, adverse events, particularly statin-associated muscle symptoms, are reported in a reduced percentage of users and have been suggested to be linked to mitochondrial dysfunction (Zhang et al. 2022).

The clinical management of dyslipidemia has been standardized by global guidelines, such as those from the American Heart Association and the European Society of Cardiology, which emphasize statins as the first-line therapy for reducing atherosclerotic cardiovascular disease risk (Grundy et al. 2019). Statin therapy is most prevalent in middle-aged and older adults, populations often characterized by a gradual decline in physiological reserve; therefore, understanding the systemic impacts of these drugs beyond lipid lowering is a public health priority (Zhao et al. 2015). While the cardiovascular benefits are indisputable, the high volume of prescriptions necessitates a rigorous evaluation of potential secondary effects on highly metabolic organs, such as the brain (Grundy et al. 1990; Li et al. 2019; Zhang et al. 2022).

Emerging evidence suggests that atorvastatin’s impact on mitochondrial function is tissue-dependent (Christiansen et al. 2021). While it may impair respiration and ATP production in skeletal muscle and liver (Li et al. 2019; Zhang et al. 2022), studies in cardiac and brain tissues report both protective and detrimental outcomes (Zheng et al. 2021, 2023). Notably, a recent in vitro investigation on isolated rat brain mitochondria revealed that direct exposure to atorvastatin dose-dependently inhibits respiratory chain activity and oxidative phosphorylation efficiency (Wojcicki et al. 2024).

In contrast, we previously showed that atorvastatin treatment (10 mg/kg, for 7 consecutive days) increased mitochondrial function and respiratory capacity in the mouse hippocampus. Such an effect was observed even after the infusion of the neurotoxic β-amyloid peptide (Mancini et al. 2020). Additionally, we have demonstrated that this same treatment protocol protects the hippocampus against ex vivo ischemic-like injury by preventing the reduction of glutamate uptake and glutamine synthetase activity through the modulation of oxidative stress (Vandresen-Filho et al. 2013). Furthermore, atorvastatin administration reversed depressive-like and anhedonic-like behaviors induced by β-amyloid peptide infusion, an effect associated with increased cleavage of proBDNF into mature BDNF in the hippocampus (Ludka et al. 2017a).

Atorvastatin is a lipophilic compound, a property that facilitates its crossing of the blood-brain barrier and allows for direct interaction with CNS cells (Liao and Laufs 2005; Ludka et al. 2017a). This penetration into the brain parenchyma is a double-edged sword: while it enables the pleiotropic neuroprotective and antioxidant effects observed in various models of neurodegeneration (Barone et al. 2014; Marques et al. 2018b; Mancini et al. 2020), it also raises questions about potential interference with brain-specific cholesterol pools (Björkhem et al. 2004). Since the brain is the most cholesterol-rich organ in the body and relies heavily on precise lipid homeostasis for synaptic integrity and mitochondrial dynamics, the regional sensitivity of different cortical areas to lipophilic statin exposure remains a critical point of scientific inquiry (Christiansen et al. 2021; Wojcicki et al. 2024).

Although statins’ effects on the hippocampus have been frequently addressed (Piermartiri et al. 2009; Martins et al. 2015; Ludka et al. 2017b), little is known about atorvastatin’s effects on the cerebral cortex, a region critical for higher cognitive functions and usually affected in neurodegenerative disorders (Aggleton et al. 1999; Ranganath 2006; Norman 2010). To address this literature gap, we investigated whether short-term atorvastatin administration, in the same protocol we previously showed neuroprotective effects (Martins et al. 2015; Ludka et al. 2017a; Mancini et al. 2020), may alter mitochondrial respiration and redox status in cortical tissue from adult mice under basal conditions.

## Materials and Methods

### Animals and Treatment

**Fig. 1:**
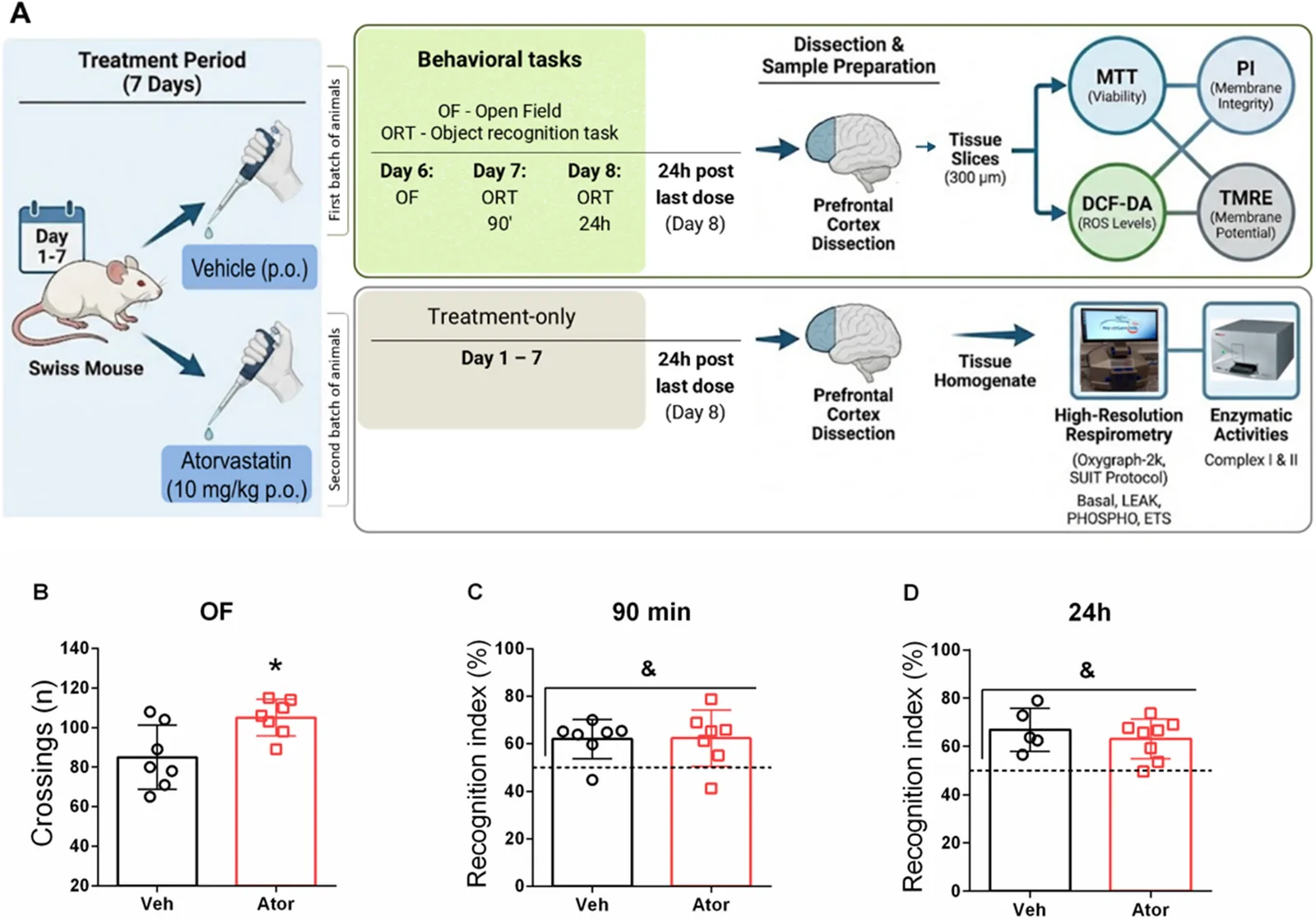
Experimental design and behavioral analysis of short-term atorvastatin administration. (**A**) Schematic representation of the experimental protocol. Swiss mice received atorvastatin (10 mg/kg, p.o.) or vehicle for 7 consecutive days. Behavioral tasks were performed on day 6 (Open Field test), day 7 (Novel Object Recognition task, 90 min retention), and day 8 (Novel Object Recognition task, 24 h retention). (**B**) Locomotor activity evaluated by the total number of crossings in the Open Field test. (**C**) Short-term memory was assessed by the discrimination index in the Novel Object Recognition task (ORT, 90 min interval). (**D**) Long-term memory was assessed by the discrimination index in the Novel Object Recognition task (24 h interval). Data are presented as mean ± S.E.M. (*n* = 6–8 per group). In (**B**), * *p* < 0.05 compared to the Vehicle group (Student’s t-test). In (**C**) and (**D**), & *p* < 0.05 compared to the theoretical chance level of 50% (one-sample t-test), indicating significant object recognition

Male adult Swiss albino mice (3 months old/ 45–55 g) were kept on a 12-h light/dark cycle (light on at 07.00 a.m.) at a constant temperature of 22 ± 1 °C. According to established standards in biomedical research, 3-month-old mice are developmentally comparable to humans aged approximately 20–30 years, providing a stable physiological baseline for mechanistic investigations (Flurkey et al. 2007; Dutta and Sengupta 2016). Mice were housed in plastic cages with tap water and commercial food ad libitum. All experimental procedures were approved by the Institutional Animal Care and Use Committee of the Federal University of Santa Catarina (CEUA-UFSC − 7173060520) and conducted in accordance with national guidelines for the care and use of laboratory animals. Mice were randomly assigned to receive vehicle (Veh) or atorvastatin (Ator) once daily for 7 days. To ensure unbiased results, all behavioral scoring and biochemical assays were performed by researchers blinded to the treatment allocation. For all experiments, N represents the number of individual animals per group (6–8). Experiments were carried out in two experimental sets: (i) mice used for behavioral analysis, followed by ex vivo cortical slices preparation and probes analysis in triplicate, and (ii) mice used for preparation of cortical homogenates (fresh tissue) for high-resolution respirometry (HRR) and enzymatic activity measurements (frozen homogenates), as depicted in Fig. 1.

Atorvastatin (10 mg/kg/day) was orally administered (p.o., in a voluntary consumption protocol) by using a palatable solution composed of condensed milk diluted in filtered water (in a ratio of 1:10, Vehicle). The experimenter was previously trained by a veterinary physician to ensure the safety and efficacy of the micropipette-guided administration. Consumption of the full dose was visually confirmed for each animal. Dosing was performed consistently every morning between 9:00 and 10:00 am to minimize circadian variation. The dose of 10 mg/kg/day was selected based on its previously demonstrated neuroprotective effects in rodent models (Mancini et al. 2020). To ensure clinical relevance, the Human Equivalent Dose (HED) was estimated using body surface area normalization according to the formula: HED (mg/kg) = Animal dose (mg/kg) times (K_m_ animal/ K_m_ human) (Reagan-Shaw et al. 2008). Considering K_m_ factors of 3 for mice and 37 for a 70-kg adult, 10 mg/kg in mice corresponds to approximately 0.81 mg/kg in humans (57 mg/day), which falls within the FDA-approved therapeutic range for atorvastatin (10–80 mg/day) (Pfizer Inc. 2019).

Mice were previously trained to ingest the solution through a 200-microliter micropipette until it was no longer necessary to restrain the animal to reduce handling and stress (Scarborough et al. 2020). The volume administered did not exceed 200 µl, according to mice’s weight. Control mice received an equivalent volume of vehicle solution. The weight of the animals was monitored during the treatment, and no changes were observed due to the ingestion of the palatable vehicle.

### Behavioral Task

Tasks were performed between 10:00 am and 5:00 pm under low-intensity light (12 lx) following a 1-hour habituation. Sessions were video-recorded and manually analyzed.

#### Open-field (OF) Test

The locomotor activity of mice was evaluated using an open-field task. The apparatus consisted of a wooden square arena (40 × 60 × 50 cm). The floor of the arena was divided into 12 equal squares. Mice were placed individually in the center of the arena, and their movement was recorded. The number of squares crossed with all paws (crossings) was counted in a 5-minute session. The light was maintained at reduced intensity to avoid anxiety behavior (Netto et al. 1986).

#### Novel Object Recognition Task (ORT)

Performed 24 h later in the same apparatus, the novel object recognition task consisted of two 5-min sessions (training and test) separated by a 90-min (short-term memory) and 24 h interval (long-term memory). The object identity (e.g., glass vs. plastic) and location were counterbalanced across animals to prevent inherent preference or side bias. Between each trial and animal, the arena and objects were thoroughly cleaned with 70% ethanol to eliminate olfactory cues. During training, mice explored two identical objects; in the test period, one was replaced by a novel object. Exploration was defined as sniffing or touching the object at a distance < 2 cm (Leger et al. 2013). The discrimination ratio was calculated as TN / (TN + TF) × 100, where TN is the time exploring the novel object, and TF is the time exploring the familiar object. Results are expressed as a percentage of recognition index and compared to the theoretical chance level of 50% (one-sample t-test), indicating significant object recognition.

### Preparation of Prefrontal Cortices Slices

Mice were euthanized by decapitation 24 h after the last day of treatment. The brain was removed, and the prefrontal cortices were rapidly dissected in ice-cold Krebs-Ringer bicarbonate buffer (KRB; of the following composition: 122 mM NaCl, 3 mM KCl, 1.2 mM MgSO_4_, 1.3 mM CaCl_2_, 0.4 mM KH_2_PO_4_, 25 mM, NaHCO_3_, and 10 mM D-glucose). Prefrontal cortices were sliced into 300 μm thick transverse sections with a McIlwain tissue chopper. Slices were then transferred to and separated into an ice-cold KRB bath (Molz et al. 2008).

### Cellular Viability and Fluorescence Assays

The effects of atorvastatin treatment on prefrontal cortex slices were evaluated by assessing cellular viability, oxidative stress, cell membrane integrity, and mitochondrial membrane potential. Cellular viability was determined using the MTT reduction assay. Slices were incubated with MTT (0.5 mg/mL) for 20 min at 35 °C. The solution was withdrawn and discarded, the precipitated formazan was solubilized with 200 µl DMSO, and absorbance was measured at 550 nm in a TECAN^®^ microplate reader. Results were expressed as optical density (O.D.).

For fluorescence assays, ROS production, cellular membrane integrity, and mitochondrial membrane potential were assessed using the probes 2′,7′-dichlorofluorescein diacetate (DCFH-DA; 80 µM), propidium iodide (PI; 7 µg/mL), and tetramethylrhodamine ethyl ester (TMRE; 100 nM), respectively. Slices were incubated with the probes for 30 min at 35 °C. After washing with KRB, fluorescence was quantified using a TECAN^®^ microplate reader at the following excitation/emission wavelengths: 480/525 nm for DCFH-DA, 495/630 nm for PI, and 550/590 nm for TMRE. Results were expressed as relative fluorescence units (RFU). All assays were performed in triplicate slices for each experimental group.

### Preparation of Prefrontal Cortices Homogenates for Mitochondrial Function

For the evaluation of the mitochondrial O_2_ consumption, after dissection, fresh prefrontal cortex tissue was weighed and homogenized using a glass homogenizer in 500 µl of sucrose-rich buffer (“respiration buffer”), pH 7.4, containing 320 mM sucrose, 1 mM EGTA, 4 mM MgCl_2_, 5 mM KH_2_PO_4_, and 10 mM Tris-HCl. The homogenate was kept on ice for immediate use (Liu et al. 2024).

### High-Resolution Respirometry

Mitochondrial oxygen consumption was measured using an Oxygraph-2k (Oroboros Instruments, Innsbruck, Austria) following a substrate–uncoupler–inhibitor titration (SUIT) protocol. Fresh cortical homogenates (2 mg/mL) were added to 2 mL respiration buffer at 37 °C under constant stirring. The O_2_ consumption rate (OCR) was determined by the mass of tissue (pmol O_2_/s/mg), measured in real-time using DatLab software (Oroboros Instruments, Innsbruck, Austria), as described previously (Burtscher et al. 2015). Following stabilization and basal OCR, LEAK respiration was induced with pyruvate (5 mM), malate (0.5 mM), and glutamate (10 mM). Complex I (CI) oxidative phosphorylation was stimulated by ADP (0.5 and 1 mM), followed by succinate (10 mM) to support CI&II-linked respiration. Maximal ETS capacity was determined by FCCP titration (0.2–0.5 µM). Finally, residual oxygen consumption (ROX) was measured after adding rotenone (0.5 µM) and antimycin A (2.5 µM), oxygen consumption rates were corrected for ROX by subtracting the non-mitochondrial respiration measured after antimycin A addition (Amoêdo et al. 2011). Detailed protocol is presented in Table 1. The respiratory control ratio (RCR) and spare respiratory capacity (SPARE) were calculated as described previously (Gnaiger 2007).

**Table 1:**
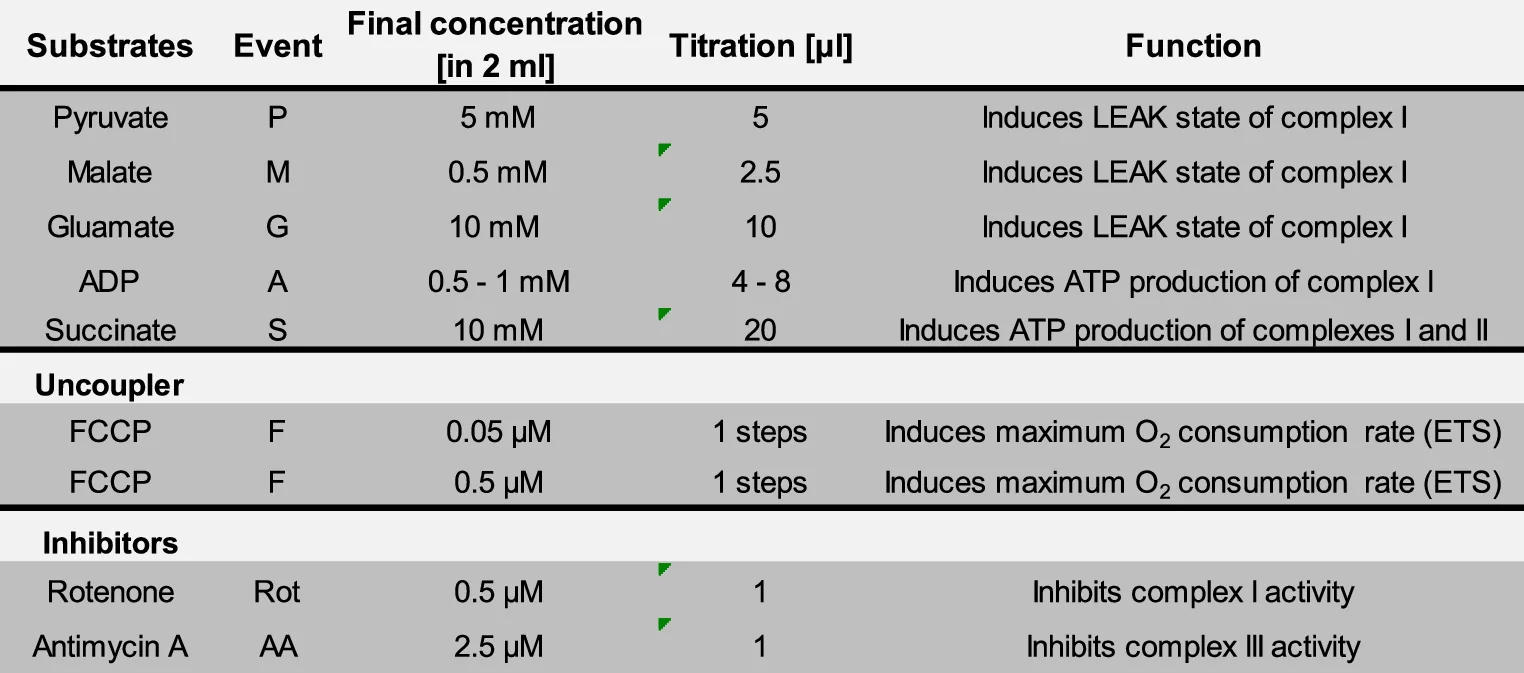
Substrate-Uncoupler-Inhibitor-Titration (SUIT) protocol for High-Resolution Respirometry (HRR)

### Determination of the Respiratory Chain Enzyme Activities

Complex I (NADH dehydrogenase) activity was determined by measuring the rate of NADH-dependent ferricyanide reduction, as previously described by Cassina and Radi by (1996). Complex II (succinate dehydrogenase) activity was assessed by measuring the succinate-dependent reduction of 2,6-dichloroindophenol (DCIP), according to the method described by Fischer et al. (1985). The methods described were slightly modified, as detailed in a previous report (Latini et al. 2005). Enzymatic assays were performed at 37 °C in a sucrose-rich buffer described above (pH 7.4), and results were normalized by total protein content determined via the Lowry method. The activities of the respiratory chain complexes were calculated as nmol/min/mg protein.

### Statistical Analysis

Data are expressed as mean ± standard error of the mean (SEM). Statistical significance was determined using unpaired two-tailed Student’s t-test. The ORT task was analyzed by one-sample *t-test* to determine whether the recognition index was different from 50% (random investigation). A p-value < 0.05 was considered statistically significant. All tests were performed using the GraphPad Prism 6.0 software package.

## Results

### Behavioral Assessment

Figure 1A schematically depicts the treatment of animals and the two experimental sets: (i) used for behavioral analysis, followed by ex vivo cortical slices preparation, and (ii) used for preparation of cortical homogenates for high resolution respirometry (HRR) and enzymatic activity measurements.

Behavioral evaluation in the open field test showed that atorvastatin-treated mice (10 mg/kg, for 7 days) exhibited increased (t(13) = 3.185, *P* = 0.0072) spontaneous locomotor activity, as indicated by a higher number of crossings compared to vehicle-treated animals (Fig. 1B). In the novel object recognition task, both vehicle- and atorvastatin-treated groups showed recognition index values significantly above the chance level at 90 min (t(6) = 3.872, *P* = 0.0082 and t(6) = 2.757, *P* = 0.0330, respectively) and at 24 h (t(4) = 4.241, *P* = 0.0130 and t(7) = 4.522, *P* = 0.0027, respectively) (Fig. 1C and D). These results indicate that recognition memory was not altered at either time point in both short- and long-term retention trials.

### Atorvastatin Does Not Affect Cortical Redox Status or Cell Viability

**Fig. 2:**
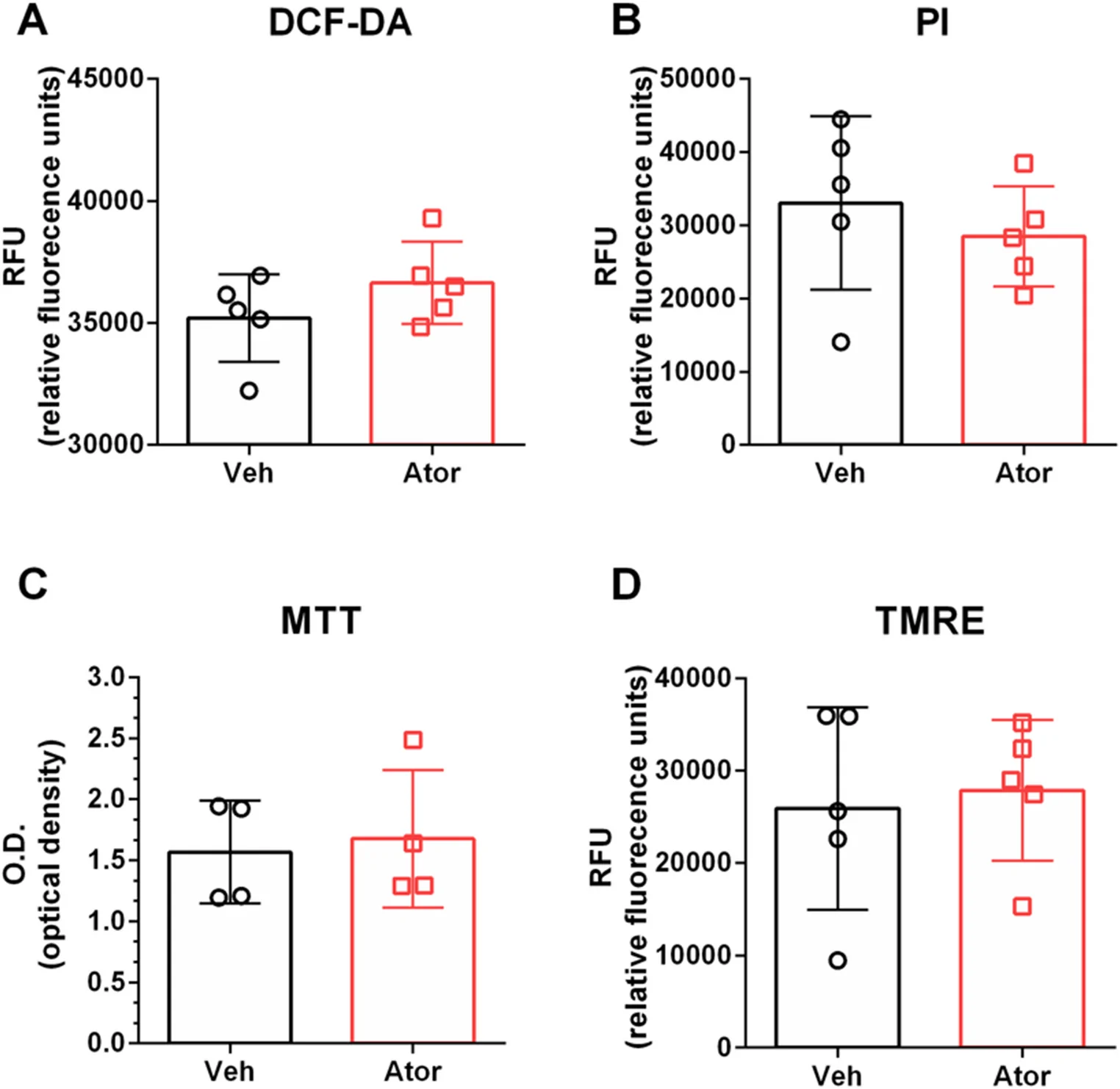
Effect of short-term atorvastatin treatment on cellular viability and mitochondrial parameters in prefrontal cortex slices. (**A**) Intracellular reactive oxygen species (ROS) production measured by DCF-DA fluorescence. (**B**) Evaluation of cell membrane damage using Propidium Iodide (PI) uptake. (**C**) Cellular metabolic viability assessed by the MTT reduction assay. (**D**) Mitochondrial membrane potential assessed by TMRE fluorescence. Bars represent mean ± S.E.M. (*n* = 4–6 independent experiments carried out in triplicate). No significant differences were observed between groups (Student’s t-test)

To investigate the effects of atorvastatin on prefrontal cortical slices` homeostasis, we first assessed key markers of cellular health and stress. Following in vivo atorvastatin treatment (10 mg/kg, for 7 days), we observed no significant changes in the levels of reactive oxygen species (ROS) (t(8) = 1.312, *P* = 0.2261), as measured by DCF-DA fluorescence in slices obtained from prefrontal cortex (Fig. 2A). Furthermore, atorvastatin did not induce cell membrane damage (t(8) = 0.7437, *P* = 0.4783), evidenced by a lack of propidium iodide (PI) uptake (Fig. 2B), nor did it compromise overall metabolic viability (t(6) = 0.3102, *P* = 0.7669), as determined by the MTT reduction assay (Fig. 2C). Consistent with this lack of cytotoxicity, the mitochondrial membrane potential, a critical indicator of mitochondrial health, was also not compromised in the atorvastatin-treated slices (t(8) = 0.3269, *P* = 0.7522), as measured by TMRE fluorescence (Fig. 2D). Taken together, these findings suggest that atorvastatin does not induce oxidative stress, cytotoxicity, or mitochondrial depolarization in the prefrontal cortex under these conditions.

### Cortical Mitochondrial Respiration is not Altered by Short-term Atorvastatin Treatment

**Fig. 3:**
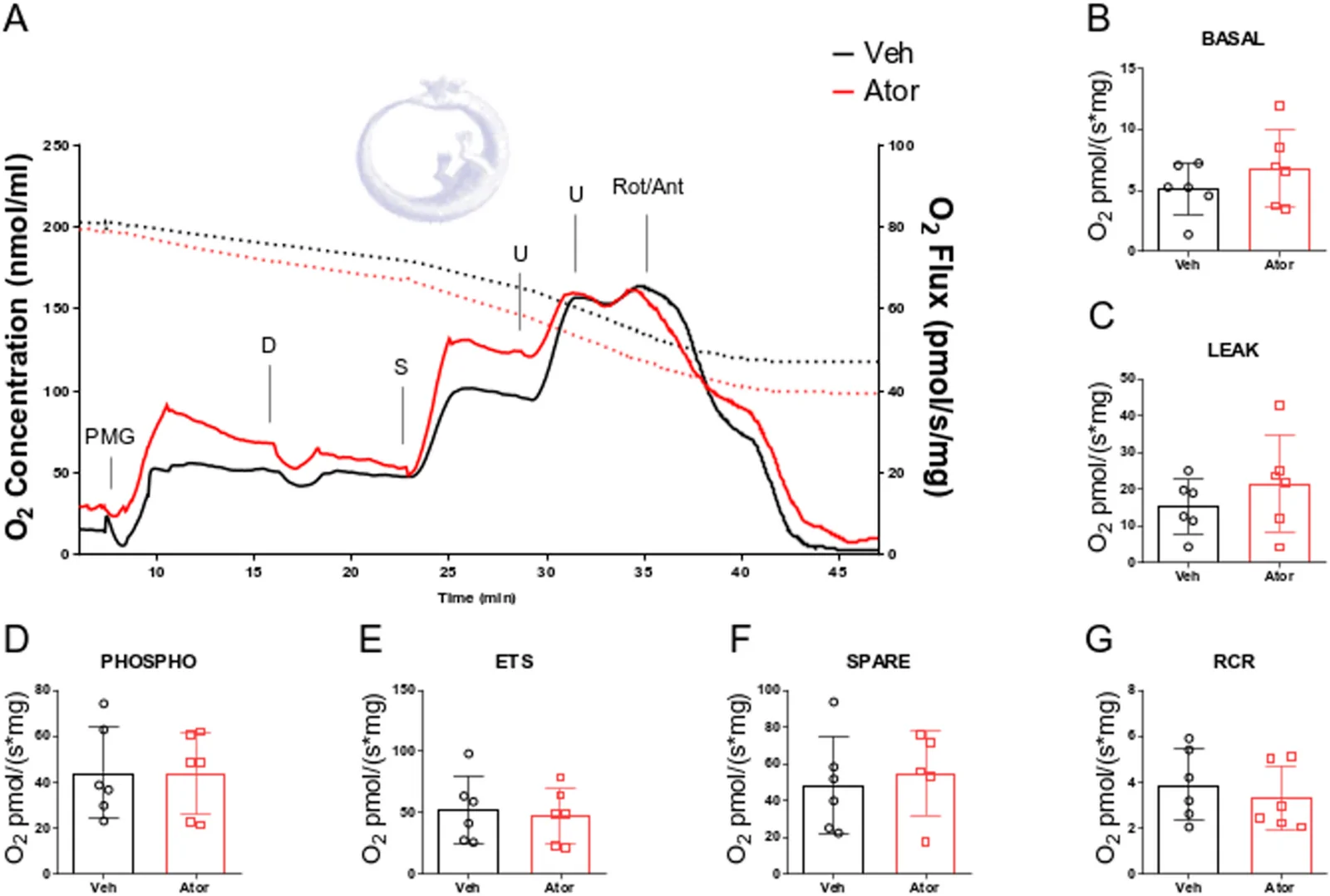
Mitochondrial respiratory function in prefrontal cortex homogenates assessed by high-resolution respirometry. (**A**) Representative oxygraphic trace showing oxygen concentration (dotted line) and oxygen flux (solid line) during the SUIT protocol. The arrows indicate the sequential addition of substrates and inhibitors: PMG (Pyruvate + Malate + Glutamate), A (ADP), S (Succinate), F (FCCP), Rot (Rotenone), and AA (Antimycin A). Concentrations and volumes are detailed in Table 1. Quantitative analysis of Oxygen Consumption Rates (OCR) in different respiratory states: (**B**) Basal respiration, (**C**) LEAK state, (**D**) Phosphorylating respiration (PHOSPHO), and (**E**) Maximal Electron Transport System capacity (ETS). (**F**) Spare respiratory capacity. (**G**) Respiratory Control Ratio (RCR). Data are expressed as pmol O₂/(s·mg) tissue and were corrected for residual oxygen consumption (ROX). Bars represent mean ± S.E.M. (*n* = 6). No significant differences were observed (Student’s t-test)

Mitochondrial integrity is crucial for neuronal homeostasis, and its disruption is a key mechanism of potential drug-induced toxicity. Therefore, we assessed the effects of atorvastatin on mitochondrial respiration in prefrontal cortex crude homogenates using high-resolution respirometry (HRR) to evaluate O₂ consumption rates (OCR). Figure 3A shows a representative oxygraphy trace of a full substrate-uncoupler-inhibitor titration (SUIT) protocol. This protocol (detailed in Table 1) allowed us to determine several respiratory states: LEAK respiration, supported by Complex I substrates (pyruvate, malate, and glutamate, PMG); PHOSPHO, or ADP-stimulated phosphorylating respiration which is initiated by the addition of ADP (A); and maximal Electron Transport System (ETS) capacity, obtained by uncoupling the respiratory chain with the ionophore FCCP. Residual non-mitochondrial O₂ consumption was determined after blocking Complexes I and III with rotenone and antimycin A, respectively. As shown in Fig. 3, atorvastatin treatment had no significant impact on the OCR values. Specifically, no differences were observed between the vehicle- and atorvastatin-treated groups for basal respiration (t(10) = 1.119, *P* = 0.2891) (Fig. 3B), LEAK respiration (t(10) = 1.028, *P* = 0.3284) (Fig. 3C), PHOSPHO state (t(10) = 0.0211, *P* = 0.9836) (Fig. 3D), or maximal ETS capacity (t(10) = 0.3537, *P* = 0.7309) (Fig. 3E). Furthermore, the mitochondrial spare respiratory capacity, which is the capacity of mitochondria to increase electron flow in situations of increased energy demand, was not altered by atorvastatin (t(9) = 0.4240, *P* = 0.6816) (Fig. 3F). The respiratory control ratio (RCR), a measure of coupling efficiency, also remained unchanged (t(10) = 0.6878, *P* = 0.5072) (Fig. 3G). Overall, atorvastatin treatment did not significantly impact the OCR parameters evaluated in the prefrontal cortex.

### Cortical Mitochondrial Complex Respiratory I and II activity is Not Altered by Short-term Atorvastatin Treatment

To complement the high-resolution respirometry data, we evaluated the specific enzymatic activities of the electron transport chain complexes that comprise the two entry points into oxidative phosphorylation, complex I and II. We observed that atorvastatin treatment did not induce any significant alteration in the enzymatic activity of Complex I (NADH dehydrogenase) compared to the vehicle group (Table 2). Similarly, the activity of Complex II (succinate dehydrogenase) remained unchanged following the short-term administration of atorvastatin (Table 2). These results reinforce the observation that the functional integrity of the electron transport chain components is not compromised in the prefrontal cortex following atorvastatin treatment.

**Table 2:**
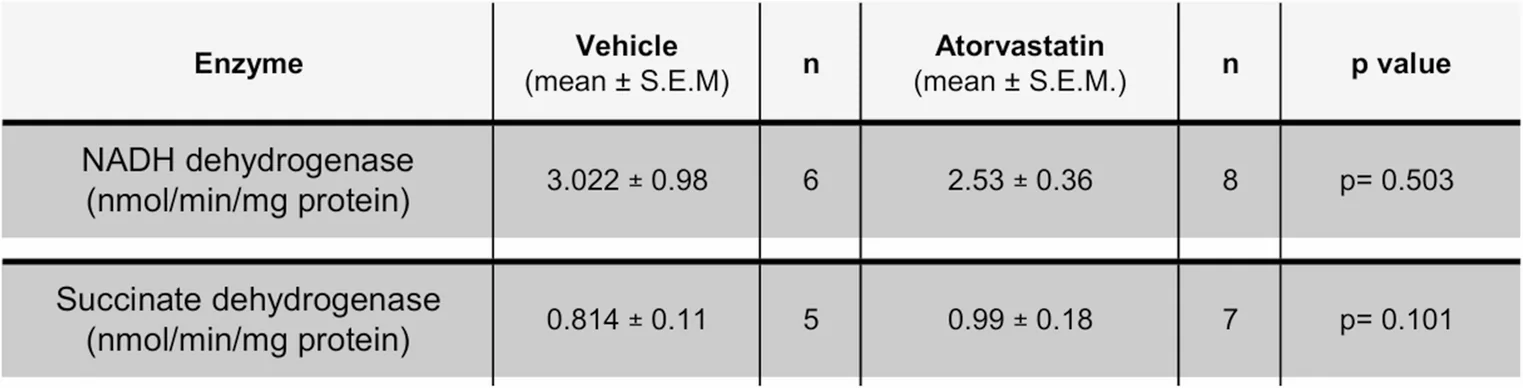
Specific enzymatic activities of mitochondrial respiratory chain complexes I and II in homogenate from the mouse prefrontal cortex

## Discussion

The main finding of this study is that *in vivo* short-term atorvastatin treatment exerts a neutral effect on cellular and mitochondrial homeostasis of the mouse prefrontal cortex. This stands in contrast to numerous reports demonstrating that statins can alter mitochondrial bioenergetics in other tissues, such as skeletal muscle (Zhang et al. 2022) and liver (Li et al. 2019). Specifically, our analysis demonstrated that atorvastatin did not impair cellular viability, cell membrane integrity, redox status, and mitochondrial membrane potential. This absence of cellular stress was confirmed by high-resolution respirometry and specific enzymatic assays, which showed no alterations in oxygen consumption, coupling efficiency, or Complex I and II activities. These data indicate that atorvastatin, at this dose and duration, does not directly inhibit electron transport chain components nor mask latent defects through compensatory mechanisms. This atorvastatin protocol of treatment in preclinical studies (10 mg/kg p.o., for 7 days) was initially shown to be neuroprotective in a trauma brain injury rodent model (Lu et al. 2004). Then, this therapeutically effective dosage in humans was also tested in rodent models of several brain diseases (Lu et al. 2004; Martins et al. 2015; Ludka et al. 2017b; Mancini et al. 2020; Zheng et al. 2021), showing neuroprotective effects against oxidative damage and glutamatergic excitotoxicity, mainly by evaluating the hippocampus.

Regarding the translational relevance, the use of 3-month-old mice represents a mature adult stage, roughly equivalent to humans aged 20–30 years (Flurkey et al. 2007; Dutta and Sengupta 2016). While statin therapy is clinically more prevalent in older populations, this model allowed us to isolate the drug’s impact on healthy cortical bioenergetics without the confounding influence of age-related mitochondrial decline. Although we acknowledge the lack of direct pharmacokinetic or pharmacodynamic markers in the present study, this specific protocol has been previously shown to reach the CNS and exert neuroprotective effects (Castro et al. 2013; Marques et al. 2018a). In the clinical context, patients with cardiometabolic comorbidities or advanced age may present a pre-existing mitochondrial vulnerability that could differ from our healthy young-adult model. Therefore, while our results demonstrate a safety profile under basal conditions, future studies should explore whether this neutrality persists in the aging brain.

Despite the absence of detectable mitochondrial or cellular alterations in the prefrontal cortex, atorvastatin treatment was associated with the maintenance of functional cognitive performance. Specifically, the results from the novel object recognition task showed that treated animals retained their ability to discriminate between familiar and novel objects at levels comparable to the vehicle group. Since our study employed healthy young-adult mice, which already exhibit optimal baseline performance, the absence of group differences indicates that atorvastatin is behaviorally neutral and does not impair memory formation or retrieval under basal conditions.

These findings contrast with studies showing that high-dose atorvastatin impairs mitochondrial respiration and increases oxidative stress in skeletal muscle and liver (Li et al. 2019; Zhang et al. 2022). On the other hand, recently a clinical study showed that treatment with 20 mg/kg of atorvastatin for 12 weeks did not alter the expression of genes related to mitochondrial, oxidative, insulin, lipid, and fibrogenic pathways in the muscle of patients with diabetes mellitus. These results reinforce the safety of atorvastatin in patients with diabetes (Borges et al. 2024). Our findings of bioenergetic neutrality in the healthy prefrontal cortex are particularly relevant when considering clinical vulnerability. In elderly patients or those with cardiometabolic comorbidities, pre-existing mitochondrial dysfunction could potentially sensitize tissues to statin-associated side effects, such as myopathy. Therefore, the absence of mitochondrial impairment observed here provides a baseline safety threshold, though future research should investigate whether this neutrality persists in models of pre-existing mitochondrial vulnerability or advanced age.

In the CNS, considering the heterogeneity of brain regions, the effects of atorvastatin must be addressed and may be region-dependent. For example, we previously demonstrated that atorvastatin treatment in the same protocol evaluated herein improves hippocampal mitochondrial function, increasing oxidative phosphorylation and reducing ROS and nitric oxide levels induced by amyloid-beta peptide (Piermartiri et al. 2009; Martins et al. 2015; Mancini et al. 2020). In this context, the behavioral effects observed here are consistent with previous evidence of functional benefits of atorvastatin in hippocampal-dependent tasks, even when mitochondrial parameters in other cortical regions remain unchanged. However, it is crucial to distinguish between the absence of toxicity observed here and neuroprotection. While our data demonstrates bioenergetic neutrality in the healthy prefrontal cortex, this safety profile is a fundamental prerequisite for therapeutic application. It suggests that atorvastatin does not disrupt basal mitochondrial function, a vital feature for a drug intended to treat chronic conditions without exacerbating bioenergetic deficits in non-target brain regions. Conversely, an in vitro study showed that atorvastatin can inhibit complex I and III activities in isolated brain mitochondria, leading to bioenergetic collapse and increased oxidative burden. The authors attribute the main deleterious effect to calcium ions present in calcium-containing atorvastatin effect in vitro (Wojcicki et al. 2024).

The absence of harmful effects observed in the prefrontal cortex in our current study may reflect a scenario closer to a translational effect, considering the route of administration, the metabolism of atorvastatin, and its effects in an experimental animal, and not just in an isolated mitochondrial preparation. In our study, the drug was delivered orally via a voluntary ingestion protocol, which more closely mimics clinical conditions and avoids the stress associated with invasive routes. Furthermore, the treatment lasted only 7 days and was conducted in healthy animals under basal conditions.

Therefore, some limitations of this study should be pointed out. One of them is the exclusive use of young adult mice. While statin therapy is clinically more prevalent in middle-aged and older populations, this initial mechanistic study was designed to isolate the drug’s effects on healthy biological systems, avoiding the confounding variables associated with biological aging. By focusing on this life stage, we established a bioenergetic safety profile in the prefrontal cortex without the interference of age-related mitochondrial decline. Furthermore, only male mice were used in this study to compare with our previous hippocampal findings and to establish a stable physiological baseline for this initial cortical investigation. However, we acknowledge that these results may not generalize to females, and future studies should incorporate sex as a biological variable. Additionally, our assessment of mitochondrial membrane potential and oxygen consumption rates provides a functional readout of the bulk mitochondrial population in the prefrontal cortex. However, these techniques do not allow the evaluation of mitochondrial morphology, network architecture, or cell-type-specific responses (neuronal vs. glial). While our functional data showed no impairment, future studies employing high-resolution imaging are necessary to confirm if mitochondrial ultrastructure remains equally unaffected. Although our HRR protocol assessed maximal respiratory capacity, the tissue itself was not subjected to a ‘stress challenge’ during the treatment period. Therefore, future investigations employing a mitochondrial challenge protocol, such as subjecting the tissue to mild oxidative stress or increased metabolic demand, as we previously assessed in the hippocampus (Mancini et al. 2020), could reveal cortical bioenergetic responses that are not apparent in the absence of a secondary stressor. From a neurotoxicological perspective, these findings are particularly relevant when considering vulnerable populations. While atorvastatin appears safe in the healthy adult brain, the bioenergetic threshold for toxicity may be lower in conditions characterized by pre-existing mitochondrial impairment, such as aging, neurodegenerative diseases, or metabolic stress. Therefore, future studies should investigate whether the bioenergetic neutrality observed here in the prefrontal cortex persists in systems with compromised mitochondrial reserve capacity, where the susceptibility to drug-induced mitochondrial dysfunction might be amplified, as we previously observed in the hippocampus (Mancini et al. 2020). In summary, our conclusions apply specifically to healthy, young-adult mice under basal conditions and a short-term (7-day) exposure window, which addressed acute tolerability and safety. Whether chronic treatment or pathological states (e.g., aging or neurodegeneration) could reveal latent cortical vulnerabilities remains to be elucidated.

Concluding, our data indicate that atorvastatin administration is bioenergetically neutral in the murine prefrontal cortex, different from the improving bioenergetic capacity we previously observed in the hippocampus, within the same context of short-term (7 days) and specific dosage (10 mg/kg) regimen evaluated here. These findings contribute to the growing body of literature indicating that the effects of statins on mitochondria are highly tissue- and context-dependent, offering beneficial effects to brain tissue. Thus, while no acute cortical toxicity was observed, these results should not be generalized to long-term clinical safety profiles. Future studies are warranted to evaluate whether pathological conditions or chronic treatment regimens could reveal latent effects of atorvastatin on cortical mitochondria.

## Data Availability

Data will be made available on request.
